# Polymer design for solid-state batteries and wearable electronics

**DOI:** 10.1039/d4sc02501f

**Published:** 2024-06-13

**Authors:** Kieran G. Stakem, Freddie J. Leslie, Georgina L. Gregory

**Affiliations:** a Chemistry Research Laboratory, University of Oxford 12 Mansfield Road Oxford OX1 3TA UK georgina.gregory@chem.ox.ac.uk

## Abstract

Solid-state batteries are increasingly centre-stage for delivering more energy-dense, safer batteries to follow current lithium-ion rechargeable technologies. At the same time, wearable electronics powered by flexible batteries have experienced rapid technological growth. This perspective discusses the role that polymer design plays in their use as solid polymer electrolytes (SPEs) and as binders, coatings and interlayers to address issues in solid-state batteries with inorganic solid electrolytes (ISEs). We also consider the value of tunable polymer flexibility, added capacity, skin compatibility and end-of-use degradability of polymeric materials in wearable technologies such as smartwatches and health monitoring devices. While many years have been spent on SPE development for batteries, delivering competitive performances to liquid and ISEs requires a deeper understanding of the fundamentals of ion transport in solid polymers. Advanced polymer design, including controlled (de)polymerisation strategies, precision dynamic chemistry and digital learning tools, might help identify these missing fundamental gaps towards faster, more selective ion transport. Regardless of the intended use as an electrolyte, composite electrode binder or bulk component in flexible electrodes, many parallels can be drawn between the various intrinsic polymer properties. These include mechanical performances, namely elasticity and flexibility; electrochemical stability, particularly against higher-voltage electrode materials; durable adhesive/cohesive properties; ionic and/or electronic conductivity; and ultimately, processability and fabrication into the battery. With this, we assess the latest developments, providing our views on the prospects of polymers in batteries and wearables, the challenges they might address, and emerging polymer chemistries that are still relatively under-utilised in this area.

## Abstract 2

Delving into the tools empowering polymer chemists to design polymers for roles as solid electrolytes, multifunctional binders and active electrode materials in cutting-edge solid-state batteries and wearable devices.

## Graphical abstract

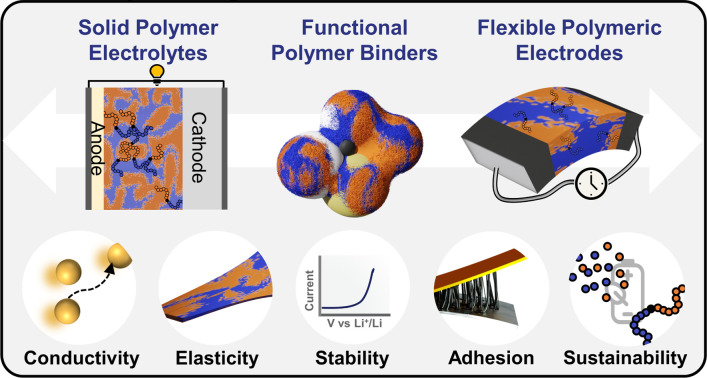

## 1. Introduction

Today, lithium-ion batteries with organic liquid electrolytes, carbon-based anodes and lithium metal oxide cathodes are the leading energy storage technology in portable electronics and electric vehicles.^1^ Since their commercialisation in 1991 by Sony, the specific energy and energy density of Li-ion batteries has more than doubled to the current state-of-the-art of >270 W h kg^−1^/>650 W h L^−1^.^2^ Alongside lower costs from mass production, these performances are largely responsible for the ‘smartphone’ era and recent global electric vehicle sales tipping >10%.^3^ Concern arises when realising how close this generation of rechargeable batteries is to their theoretical performance limits and the still pressing need for further improvements to meet net-zero energy targets.^4^ This includes inherently safer and more sustainable batteries, energy densities >1000 W h L^−1^ and specific energies >500 W h kg^−1^.^2^ Contemporary requirements are to power widespread electric vehicles for road and flying, large-scale grid energy storage for intermediate renewables (wind, solar) and bendable devices for advanced robotics, wearables, *etc.* For a detailed breakdown of battery requirements from an industry viewpoint, readers are directed to the recent publication from Ulissi and coworkers.^5^

Over the past decade, worldwide research efforts have identified several targets to increase battery performance.^6–8^ These include the development of solid-state electrolytes, broadly divided into polymers,^9^ inorganics^10^ and polymer–inorganic composites.^11^ The focus on solids is to mitigate the safety concerns associated with conventional liquid electrolytes, which are highly flammable organic carbonates.^12,13^ Solid electrolytes also potentially enable the use of high-capacity electrodes such as Li metal anodes (theoretical specific capacity of 3860 mA h g^−1^), which would provide a step change in battery energydensity.^12,14^ Originally, high-shear-moduli (*G*′) solid electrolytes in particular, were proposed to impede the growth of Li-dendrites during the charging of Li-anode batteries that lead to short-circuit and cell failure.^15–17^ In early battery commercialisation, these dendritic structures led to a significant risk of fire or explosion in liquid-based devices, resulting in anodes being restricted to Li-intercalated in graphite with a comparatively lower specific capacity of 375 mA h g^−1^.^18^

That being said, the safety credentials of the various solid electrolytes still need to be scrutinised.^19^ Some inorganic solid electrolytes (ISEs) can react with moisture to form toxic gases, and there are examples of solid polymer electrolytes (SPEs) benefiting from additives to improve their flame retardancy.^20^ Additionally, it is now known that Li dendrite growth can still occur even through mechanical stiff ISEs above certain thresholds or critical current densities.^21–23^ Low critical currents are a limiting factor in delivering practical charging times in these solid-state Li anode devices.^23^ Processing ISEs into low-cost and highly dense, conductive thin films capable of forming good interfacial contact at both electrodes is also non-trivial.^16,24^ Polymers, on the other hand, have the advantages of lightweight, tuneable mechanical performances, including high flexibility and elasticity plus advanced self-healing behaviours as well as ease of thin-film manufacture.^25,26^ Whilst stability against reactive Li-metal may still pose uncertainties, SPEs are also attractive for other next-generation electrodes such as ultrahigh capacity silicon anodes (4200 mA h g^−1^). These undergo especially large volume changes of up to 300% during charge–dischargecycles and thus require flexible electrolyte designs.^27^ Ultimately, the significant hurdle limiting SPEs is the still struggling ion transport properties. Consequently, constructing composite solid electrolytes integrating polymeric and inorganic components to improve ionic conductivity and selectively is an attractive research direction. However, this area is still not without its challenges and readers are directed to recent reviews and subsequent discussion.^28,29^

The capability of polymers to transport ions has been known since the early 1970s when salts dissolved in poly(ethylene oxide) (PEO) were discovered to form solid-ion conductors.^30^ In the last 50 years, many advances have been made in the area of polymer electrolytes (see Armand and Zhang^9^ for a perspective on their evolution), including commercialization by the Bollore group in 2010. However, the room temperature (RT) ionic conductivities of ‘dry’ (solvent-free) SPEs generally still lag behind liquids, that is, they are below 1 mS cm^−1^ compared to on the order of several mS cm^−1^ for liquid electrolytes.^31^ A distinction is made here with gel polymer electrolytes that contain large amounts of added liquid.^32^ One view is that unlocking competitive ionic conductivities of SPEs requires a re-evaluation of our current understanding of ion transport through polymers.^9^ Whilst the standard theories are based on those developed for liquids, in SPEs, charge motion is more complex.^33^ It relies firstly on dissociated ion concentrations and then the interplay of intrachain transport, interchain hopping and codiffusion of segments and ions with specific interactions, spacing of coordinating groups and dynamics of the segmental movement of the polymer backbone involved.^33,34^ Polymer chain flexibility and sequencing of ion-coordinating groups are at the helm of the polymer chemist, and this has become ever more so with developments in controlled polymer synthesis that are beginning to mimic the precision of biological systems.^35,36^ Applied, these polymerisation strategies could help in unpicking the more fundamental design rules of ion transport in polymers, but a stronger link between how measurable SPE performances truly translate into battery environments is required.

In the last 10 years, the discovery of fast-ion conducting ISEs such as sulfide-based argyrodites Li_6_PS_5_Cl puts these also *ca.* an order of magnitude more conductive than SPEs.^10^ Moreover, leading ISEs, which also include oxide-based garnets Li_7_La_3_Zr_2_O_12_ (LLZO)^37^ and halides such as Li_3_InCl_6_ ^38^ exhibit high ion selectivity, parametrised by lithium transference numbers (*t*_Li^+^_) close to unity. Despite this, solid-state batteries with ISEs have yet to be commercialised.^31^ Whilst polymers may be used to address the processing difficulties associated with ceramic brittleness,^39–41^ cathode volume changes during cycling can still cause contact losses and capacity fade that are typically only mitigated by applying unpractically high external pressures (>50 MPa).^42^ Consequently, there is a need to optimise the composite cathode microstructure for these solid-state batteries to operate under minimal external pressures (<1 MPa).^42^ Increasingly, the thought-out design of advanced multifunctional polymeric components with ion, electron, elastic, and/or mixed conducting properties offers potentially attractive solutions to this challenge.^43,44^ As polymer binders/coatings and interface modifiers, many of the design considerations of SPEs should also be applicable to their optimisation in these other roles towards commercialising ISE-based solid-state batteries.

Polymer properties are also particularly poised to deliver rechargeable batteries with the ability to bend, twist and stretch. This is increasingly relevant for wearable electronics, where the global market for flexible batteries is expected to grow by $240 million by 2027 from 2022 demand.^45,46^ Compared to powering electric vehicles, the battery requirements for wearable technologies are different; focusing more on flexibility, conformability and safety when interfaced with human skin.^47^ Generally, batteries used for health monitoring (conformable to skin) and powering flexible devices (smartwatches, foldable phones) require lower energy densities and shallower electrochemical stability windows.^48^ Many state-of-the-art polymer electrolytes and binder components could already meet these requirements, which for truly flexible devices also requires sufficiently bendable electrodes, current collectors, electrolyte separators and encapsulates. The mass marketing of wearable technologies also raises another critical aspect of electronic products – namely, concerns over battery end-of-life and the growing mountain of electronic waste.^49^ The need to design new polymeric materials with aspects of re-processability and end-of-life chemical recycling and/or degradation avenues has never been more central than for polymers in energy storage.

**Fig. 1:**
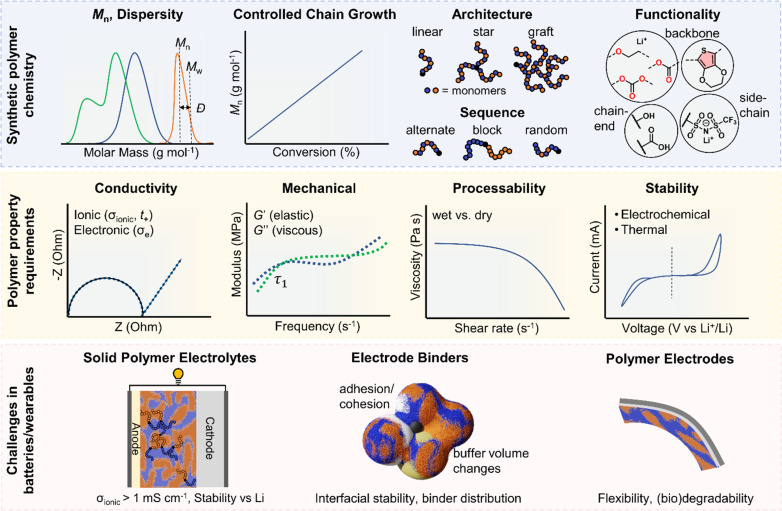
Overview of polymer design for batteries and wearable technologies. Top: The synthetic polymer chemists' domain controlling polymer molar mass (*M*_n_) and molar mass distribution (dispersity, *Ð*) through ‘living’ chain growth polymerisations that can allow access to different architectures, monomer sequences and chemical structures including backbone and pendant functionalities plus chain end-groups. Middle: Measurable properties important for polymers in batteries. Conductivity shown by a Nyquist curve from electrochemical impedance spectroscopy, mechanical properties measured by rheology (also dynamic mechanical analysis and stress–strain behaviour), processability window related to the polymer melt (dry) or electrode slurry (wet) viscosity and the electrochemical stability window measured by linear or cyclic voltammetry and thermal stability (not shown) by thermogravimetric analysis. Bottom: Challenges in solid-state and flexible batteries require (1) polymers as solid-electrolyte separators, (2) functional polymer components in composite electrodes, and (3) polymer active materials for flexible electrodes in bendable devices for wearable electronics.

In this perspective, we present a viewpoint on polymers in solid-state and flexible batteries from the tools available to polymer chemists and the challenges identified by battery scientists (Fig. 1). We consider polymers as the solid electrolyte separating anode and cathode and then as functional binder components in composite electrodes with ISEs. We also explore polymers as the bulk active material for bendable electrodes and requirements as device encapsulants in wearables. Polymer macromolecular design tools include the molar mass profile, chain growth control, monomer and block sequencing, architecture and backbone chemistries. Across the roles evaluated for polymers in batteries and wearables, polymer property requirements invariably involve a consideration of conductivity, mechanical performance, stability, adhesion, and processability/sustainability. Under each battery role, the relative importance and specific requirements for these polymer properties are used to discuss the underlying polymer design strategies and associated challenges and opportunities. This perspective primarily focuses on the still prevalent Li-based systems and particularly solid-state devices that eliminate potentially hazardous liquid components. As the field of battery technologies continues to evolve, we aim to underscore the potential significance of polymer synthetic design as a potent contributor.

## 2. Developments and challenges in solid polymer electrolytes

SPEs have been intensely explored to address the limitations of traditional liquid electrolytes in energy storage systems. Characterised by their potential to offer enhanced safety, polymers also provide considerable flexibility in design. Polymer chemical composition, molar mass (*M*_n_) and dispersity (*Ð*) of molar masses in a sample play critical roles in determining the ionic conductivity and mechanical and stability properties of SPEs (Fig. 1). Advances in synthesising block copolymers and exploring various polymer architectures have opened up new avenues for optimising these electrolytes with notable improvements in performances. Moreover, advancements in polymer chain functionalisation and single-ion conductors, which incorporate immobilised anions while allowing free movement of cations, represent promising strategies for reducing issues such as concentration polarization and improving overall efficiency. This subsection delves into the latest developments in SPE research and the formidable challenges that still persist. It also highlights sustainability as another crucial aspect that is reshaping the development of SPEs, with researchers increasingly focused on utilising bio-based and recyclable polymers to create energy storage solutions.

### 2.1. Trends in block copolymers and other architectures

**Fig. 2:**
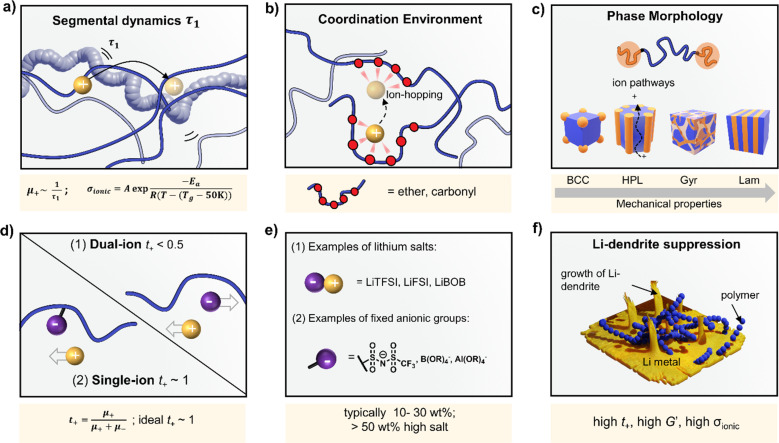
Ion transport in Solid Polymer Electrolytes (SPEs). (a) Relationships between chain segmental dynamics (*τ*_1_) and ion mobility (*μ*_+_); Vogel–Tammann–Fulcher (VTF) temperature-dependence of ion conductivity (*σ*_ionic_) with terms for polymer glass transition temperature (*T*_g_), pre-exponent *A* related to salt dissociation/free charge carrier concentrations and the activation energy for ion transport (*E*_a_). (b) Coordination environment provided by polymer backbones for controlling ion transport. (c) Phase-separated block copolymers for optimised ionic-mechanical properties. Depicted morphologies: BCC = body-centred cubic spheres, HPL = hexagonally packed cylinders, Gyr = gyroid, Lam = lamellae. (d) Dual-ion (mobile anion and cation) *vs.* single-ion (immobilised anion) conductors. Cation transference number, *t*_+_, is the ratio of cation mobility to total ion mobility. (e) Examples of common salts in SPEs and anions anchored to polymer backbones. (f) SPEs with high *σ*_ionic_, *t*_+_ ∼ 1 and 
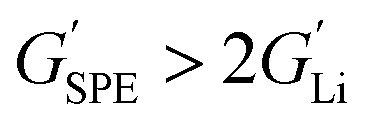
 are theorised to suppress detrimental Li dendrite growth, which currently limits Li-batteries.

Polymer ionic conductivity (*σ*_ionic_) relies on both the segmental relaxation dynamics (*τ*_1_) of polymer chains around solvated ions and the diffusion of chains that make up the surrounding ion-coordination environment (Fig. 2a and b).^34^ Originally demonstrated in PEO, cation mobility (*μ*_+_) and thus conductivity drops with increasing molar mass (*M*_n_) up until entanglement weight, where it remains constant.^50,51^ The RT Li-ion conductivities of ∼10^−5^ S cm^−1^ for PEO-based solid electrolytes are also a function of crystalline regions within the macromolecular structure, a key feature of PEO and a property to note for polymer chain organisation in general.^52,53^ These empirically substantiated relationships are important and inform controlled polymer synthesis design as *M*_n_ can be precisely targeted and crystallinity reduced. Furthermore, a decrease in polymer glass transition temperature (*T*_g_), often associated with faster segmental dynamics, can improve ionic conductivity, albeit at a loss of mechanical properties. Equally, conductivity and often mechanical performance scales with temperature and most SPEs follow a temperature-dependence of ionic conductivity that fits the Vogel–Tammann–Fulcher (VTF) equation (Fig. 2a).^54^ From this fitting of conductivity–temperature data, the apparent activation energy (*E*_a_) for ion transport (correlated to segmental dynamics) can be estimated and, in principle, decoupled from the dissociated ion content estimated from the *A* prefactor.^55^ The low RT ionic conductivity circumvented by strategies that result in poor mechanical performances, as well as its low oxidative stability, are where PEO-based electrolytes fall short. This negative correlation between mechanical robustness and ionic conductivity has led to a significant focus on designing materials to decouple the two or finding optimal compositions to balance both properties.^56^

For instance, copolymers allow for designed improvement over constituent homopolymers. Specifically, block copolymers offer a route to varying polymer phase morphologies (spheres, lamellae, *etc.*) where one microphase typically serves to conduct ions and the other to provide the desired mechanical properties (Fig. 2c). Given that softer (low *T*_g_, high *τ*_1_) polymers traditionally give higher conductivities, phase-separated block copolymers have become an attractive strategy to impart the necessary mechanical rigidity.^57^ These morphologies are highly predictable based on the block ratio, incompatibility of the chemistry (enthalpic driving force) and the overall chain length (entropic component).^58^ Although self-consistent field theory is a good approach to predicting the thermodynamic phase diagrams of block copolymers, it is recognised that for ion conductors, this may not be all-encompassing due to the presence of electrostatic interactions.^59^ Though there appears to be no unified consensus, the most desirable microphase-separated structures for ionic conductivity are those allowing for continuous ion pathways.^60,61^ Accessing these ordered morphologies for optimised ion channels significantly benefits from precisely controlled polymer synthesis. For example, Winey and coworkers were able to access the double gyroid morphology using precise ion-containing multiblock copolymers.^62^ This phase, which is attractive for ion channels, typically only represents a narrow composition region corresponding to ∼3 vol% of the predicted phase behaviours.

Aside from ion channels, a key driver behind targeting block copolymer electrolytes is the means to modify mechanical performances. For example, there has been a substantial body of work on poly(styrene)-*block*-poly(ethylene oxide), PS-*b*-PEO.^63^ With added lithium bis(trifluoromethanesulfonyl)imide (LiTFSI), PEO provides the ion-conducting ability (∼10^−5^ S cm^−1^ at RT), whilst PS confers mechanical rigidity (*G*′ ∼ 3 GPa).^64^ Ionic conductivity has been shown to decrease in a low molar mass region (2.7 and 13.7 kg mol^−1^) but increase (and eventually plateau) in a higher one (7 to 98 kg mol^−1^).^65,66^ These trends have been attributed to exclusion zones at the segmental interfaces with insulating PS; increasing *M*_n_ increases these exclusion zones, but they become negligible at sufficiently high mass. Similarly, in triblock PS-*b*-PEO-*b*-PS, conductivity was dependent on the volume fraction of PEO available, with exclusion zones now at two block interfaces.^67^ In terms of microphase separation behaviour, a range of phase morphologies have been reported, and this is influenced by salt content and most recently observed during *in situ* Small Angle X-ray Scattering (SAXS) experiments to change under applied currents.^68^

Beyond polystyrene, subsequent research has employed mechanical modifier blocks that are also capable of Li-ion coordination and conductivity. For example, di- and tri-block copolymers of PEO with polycarbonates (PC) or polythiocarbonates (PTC) from the ring-opening copolymerisation (ROCOP) of CO_2_ with 4-vinyl cyclohexene oxide (PC-*b*-PEO-*b*-PC)^69^ or COS with propylene oxide (PTC-*b*-PEO).^70,71^ For the latter, poly(propylene thiocarbonate)-*block*-PEO, the addition of LiTFSI was found to induce microphase separation due to differential interactions between the two segments and the salt, which preferentially resided in the PEO phase. This afforded a double conductive phase that offered an improved electrolyte performance to those with a single conductive phase. In the other example, poly(4-vinyl cyclohexene carbonate) (PC) was more comparable mechanically to PS, and these block copolymers also showed enhanced ionic conductivities due to dual conductive phases (0.11–0.67 mS cm^−1^ at RT) but with comparable shear-moduli (*G*′ 0.52–67 MPa).^69^ One advantage of these systems is that the second block can be initiated directly from the hydroxyl-end-groups of PEO, whereas polystyrene growth requires end-group modification first.

Controlled chain-growth techniques, including anionic/cationic olefin polymerisations, ring-opening polymerisations (ROP; anionic, cationic, radical, coordination), and those relying on reversible-deactivation processes such as reversible addition–fragmentation chain transfer (RAFT) and atom transfer radical polymerisation (ATRP) are common synthetic techniques to access block copolymers. These enable polymers with narrow molar mass distributions (*Ð* < 1.1), targeted chain lengths and a variety of well-defined monomer combinations. Many of these techniques display ‘living’ characteristics with minimal/no chain growth termination steps – allowing for chain extension through subsequent monomer additions and, thus, the construction of sequence-controlled blocks. For example, the ROP of trimethylene carbonate (TMC) and ε-caprolactone (CL) monomers can proceed to CO_2_/epoxide ROCOP when CO_2_ is introduced to yield PC-*b*-P(TMC-*co*-CL)-*b*-PC without the need for intermediate purification steps.^72^ This is also possible because the same catalyst can conduct both ROP and ROCOP mechanisms.^73^ Another approach is the use of a difunctional ATRP agent where a hydroxyl group functionality was used to initiate TMC/CL ROP to form the ion-conducting segment, and then ATRP was conducted to form a poly(benzyl methacrylate) mechanical modifier block.^74^

These features of controlled chain-growth techniques, including high chain end-group fidelity, mean that the sequencing of monomers and multiple block segments can increasingly be more precisely encoded.^36^ Future research efforts should focus on accessing these more complex block structures (ABC, pentablocks *etc.*) that might help our understanding of the complex interplay of factors that impact SPE performances.^35^ These efforts should not be limited to linear polymer architectures but also target ‘graft’ and ‘star’ structures to obtain missing correlations between or new decoupling approaches to mechanical-ionic properties.^75,76^ Again, to ensure these complex architectures are formed, controlled polymerisation strategies are needed – to initiate polymer chains and blocks sequentially from 3-, 4-, and 5-armed star species.

### 2.2. Polymer/salt combinations and single-ion conductors

Typically, the source of mobile charge carriers in polymer electrolytes is a salt dissolved in the polymer matrix (Fig. 2d and e). By far, the most commonly reported is LiTFSI due to its high thermal stability and large anion that favours salt dissociation. It should be remembered, however, that these salts can have drawbacks in terms of cost, toxicity, and reactivity with other cell components. Other common salts include lithium bis(fluorosulfonyl)imide (LiFSI), lithium bis(oxalate) (LiBOB) and lithium difluoro(oxalato)borate (LiFBOB). The type and quantity of Li salt are crucial to ion transport properties, and the development of alternatives is an ongoing research area.^77,78^

Unfortunately, most of the charge transport in these polymer/salt systems is often conducted by the anion and not the desired cation. In PEO/LiTFSI, the TFSI anion is about 4× more mobile than the highly ether-coordinated Li-cations. This results in poor selectivity for Li-ion transport expressed as a lithium transference number, *t*_Li^+^_ < 0.2.^34^ Such low values are a problem because the movement of charge in opposite directions leads to polarization and large overpotentials in the cell. These low values have been largely circumvented through high-salt loadings (>50 wt%),^25^ anion trapping strategies^79^ and covalent attachment of the anion to the polymer backbone (single-ion conductors).^80^ Such strategies, however, usually result in decreased stability for high salt systems and lower ion conductivity for the anion trapping and single-ion conductors. The reason for the latter is still not well understood.^34^ Immobilisation of the anion in single-Li-ion conductors (SLICs) generally results in *t*_Li^+^_ of 0.85 to 0.99 (Fig. 2d and e).^81,82^ This is promising as theoretical models suggest that *t*_Li^+^_ close to unity, coupled with high shear-moduli that are roughly twice that of Li anodes 
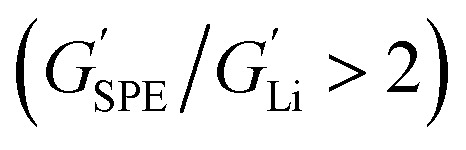
, could suppress Li-dendrite growth (Fig. 2f).^17^ Experimental evidence, however, to support or disprove this is still lacking. To add to the debate, recent claims from electrochemical polarisation studies carried out on PEO–SLICs have suggested that the advantage of single-ion conduction might only be meaningful at high currents deemed impractical.^83^

Analyses of recent reviews on SLICs provided by Gao *et al.*^81^ and Lian and coworkers,^82^ indicate that current best performances come from immobilised TFSI-like anions and, most recently, borates and aluminates. Early leading examples reported triblock polymers consisting of central PEO blocks with single-ion conducting lithium poly(styrene trifluoromethanesulphonylimide), P(STFSILi) blocks on either side.^84^ Dry, the electrolytes yielded ionic conductivities of 0.013 mS cm^−1^ at 60 °C and *t*_Li^+^_ > 0.85. Notably, an order of magnitude higher tensile strength was observed compared to the neutral structure. Similar immobilised anions were later reported on poly(methyl methacrylate) (PMMA) backbones.^85^ Results from both studies were included in a recent benchmarking of solid-state Li-metal battery performances by Janek and coworkers.^14^ They compared well to other solid-state batteries in the study, but only at temperatures above 50 °C, which were required to achieve sufficient ion conductivities.

While these findings are promising, they also underscore the need for continued research into polymeric SLICs. Several reports of TFSI-like derivatised thiols and azides offer potential avenues for advancement.^79,85,86^ These synthetic handles could provide more versatile and generalisable approaches to attaching the TFSI-structural motifs to any polymer bearing a vinyl or alkyne group using efficient thiol–ene or ‘click’ chemistry. This could facilitate the interchangeability of different polymer backbones. For instance, switching from a PMMA backbone to a softer (lower *T*_g_) polysiloxane has led to accelerated segmental dynamics in SLICs and orders of magnitude higher conductivities.^86^

Considering other anions, lower electronegativities of the central atom and more delocalised charges are preferable for higher ambient ionic conductivities. This was demonstrated recently in work that incorporated pendant lithium borate single-ion moieties into the backbone of polymethacrylate chains.^87^ More electron-withdrawing substituents around the tetrahedral borate centre elicited higher ionic conductivities up to 0.165 mS cm^−1^ at 60 °C. In addition to facilitating charge delocalisation and salt dissociation, examples of borate-based SLICs have also demonstrated widened electrochemical stability windows and eased synthesis and cost.^88,89^ Beyond borate structures, lithium aluminates have helped deliver more dynamic, adaptable mechanical behaviours of SLICs.^90^ In polymer networks, single-ion conductivity and dynamic nature increased in the order of aluminates > borates > silicates. This mirrored the weaker Al–O > B–O > Si–O bond strengths and is a promising future avenue for flexible, dynamic single-ion systems to come.

### 2.3. Integrating sustainable polymer chemistries

**Fig. 3:**
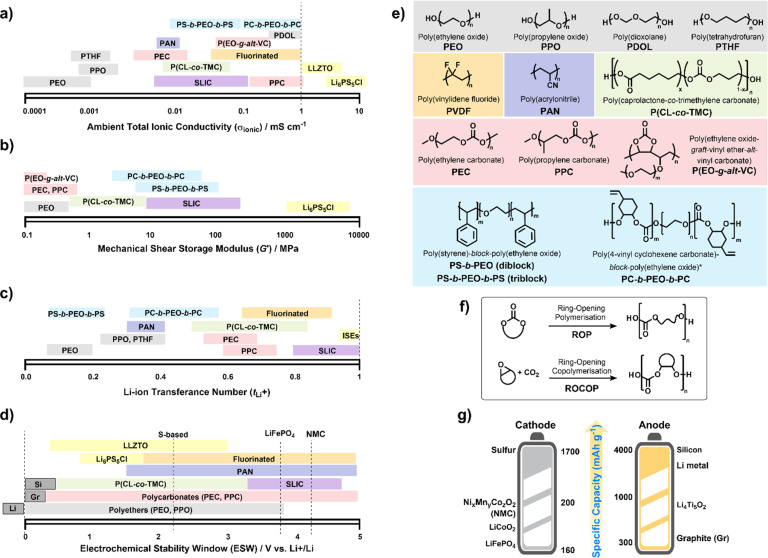
Polymer electrolyte chemistries. Properties of different families: (a) total ionic conductivities (*σ*_ionic_) measured by electrochemical impedance spectroscopy at 25–30 °C. (b) Mechanical shear-storage-moduli (*G*′) from rheological measurements. (c) Li-transference number (*t*_Li^+^_) or effective Li-ion selectivity with values sometimes differing depending on whether the Bruce–Vincent or NMR methods were used. (d) Electrochemical stability window (ESW) where operating voltages for various electrodes are indicated. (e) Electrolytes in (a–d) are categorized according to type: polyethers (grey), polycarbonates (red), fluorinated (orange), polynitriles (dark blue), polyesters (green) and triblock copolymers (blue); single Li-ion conductors SLIC (purple) include Li borates and sulfonyl imides; inorganic solid electrolytes (ISEs) in yellow include sulfide Li_6_PS_5_Cl and oxides Li_6.4_La_3_Zr_1.4_Ta_0.6_O_12_ (LLZTO). (f) Controlled polymerisation strategies to synthesise polycarbonates. (g) Theoretical specific capacities for today's leading cathode materials (*vs.* graphite anodes) and anodes (*vs.* LiFePO_4_ cathode). NB. Polymer chain ends are specified as –OH terminated but for PEO and PEC different methoxy- and acetate groups have been shown to influence (a)–(d).^91,92^ Ranges represented in (a) to (d) reflect dependence on salt, *M*_n_, *etc.* See also Table 1.

To date, numerous polymer chemistries have been investigated for their ion transport properties, electrochemical stability and mechanical performances (Fig. 3). All of these are to regulate battery performance, such as rate capability, cycling durability, and lifespan. Generally, SPEs are often limited to RT ionic conductivities below 1 mS cm^−1^ (Fig. 3a). Whereas leading oxide, halide and sulfide-based ISEs have shown ionic conductivities greater than this, particularly argyrodite-type Li_6_PS_5_Cl reaching above 10 mS cm^−1^.^93^ Clearly, identifying chemistries to improve the RT conductivities of SPEs is still critical. However, what do we expect to uncover after 50 years of research since the first discovery? Still, PEO chemistries consistently outperform others at temperatures above RT – though there have been some promising ambient conductivities reported for *in situ* formed poly(dioxolane) (PDOL) and poly(propylene carbonate) (PPC) on cellulose supports (Table 1).^96,98^

**Table 1:** Selected examples of SPEs with performances at optimal values of *M*_n_, salt (including wt% or Li : O ratio), end-group, copolymer composition and filler

Polymer^a^	*σ*_ionic_ (mS cm^−1^)	*t*_Li^+^_	*G*′^b^ (MPa)	ESW^c^ (V)	*T*_d_ d (°C)
PEO^91,94^	0.0053	0.19	0.1–1	3.9	>340
x-PTHF^95^	0.0029	0.53	2.4	4.8	234
*In situ* PDOL^96^	1.1*	—	<1	5.0	∼148
PPO/LLZO^97^	0.459	0.74	—	4.6–5.3	>250
PPC/cellulose^98^	0.3	0.2	25**	<4.6	200
PEC–Ac^92^	0.0019	0.8	—	<5.4	173
P(EO-*g-alt*-VC)^99^	0.12	0.16	—	—	339
P(CL-*co*-TMC)^100^	0.041	0.62	<0.2	0–3.5	>200
PS-*b*-PEO^65^	0.012	0.2	10	<4.5	>250
PC-*b*-PEO-*b*-PC^69^	0.23	0.62	67	4.2	>250
PAN–PEO^101^	0.68	<0.4	—	1.5–4.8	>320
LiAl(OR)_4_–FTEG^90^	0.023	0.80	∼1	∼4.2	>200
LiB(OR)_4_–PMAC^89^	0.2	0.94	0.21	4.3	122
PVDF/LLZTO^102^	0.24	0.69	12**	2–5	>225

a See Fig. 3 for chemical structures and full names. Polyethers: standard PEO/LiTFSI values; crosslinked (x) PTHF to impart mechanical performance and *in situ* converted DOL to PDOL at <100%, meaning there is some plasticisation of the chains with liquid DOL leading to enhanced conductivities (*). Polycarbonates: PPC is on cellulose support; acetate (Ac) end-capped PEC yields some enhancement over –OH terminal groups formed during synthesis, and alternating enchainment of PEO side groups, and cyclic carbonate (VC) is best with 23–24 EO repeat units. Polyester: random enchainment of 80 : 20 mol% CL : TMC is optimal and block copolymers have been explored with this mainly polyester SPE.^72,74^ Block copolymers: di-and tri-blocks reported at various compositions of PS and PEO; PC-*b*-PEO-*b*-PC is discussed later as a composite cathode binder. Single Li-ion conductors (SLIC): Li aluminate dynamic crosslinked networks with fluorinated diol, FTEG (=1*H*,1*H*,11*H*,11*H*-perfluoro-3,6,9-trioxaundecane-1,11-diol) and Li borate networks with poly(5-methyl-5-allyloxycarbonyl-trimethylene carbonate), PMAC. Fluorinated: PVDF-composites where the interface is enhanced using ionic liquid.

b Mechanical performance is not always reported, and where labelled (**) values refer to tensile strength by uniaxial stress–strain measurements rather than shear storage modulus (*G*′).

c Oxidation potential is more typically reported by linear sweep voltammetry at varying scan rates but where slower rates (0.1 mV s^−1^) are regarded as more representative.

d Onset of thermal degradation by thermogravimetric analysis (5 wt% mass loss).

In light of ISE successes, then, more research efforts might focus on investigating hybrid polymer–ISE composites.^11^ Not only are these composite electrolytes attractive to improve ionic conductivity,^103^ which can simply result from ISE fillers preventing SPE crystallinity or lowering *T*_g_ but also provide another tool to extend electrochemical stability^104^ and stiffen polymer mechanical performances (Fig. 3b and Table 1).^105^ Whilst this is a promising approach to potentially combine the ‘best of both’ solid electrolytes, there are still difficulties in the design and preparation of composite electrolytes, not least in the stability of the interfaces formed between the polymer chains and inorganic filler.^106^ More broadly, various non-conductive fillers (Al_2_O_3_, SiO_2_) have also been used to improve these SPE performance metrics.^107^ Whilst attractive, particularly with functionalised filler strategies for achieving uniform distributions,^108^ demonstrating competitive fast ion-conduction to fully dense ISEs or without soaking in liquid electrolyte remains an active target.^109^

Another key criterion for solid electrolytes is their ability to selectivity transport Li-ions. Whereas PEO may have total conductivities around 1 mS cm^−1^ above its melt (>60 °C), its low transference number (*t*_Li^+^_ ∼ 0.2) means that this corresponds to more like 0.1 mS cm^−1^ effective Li-ion conductivity. One option to overcome these ion-selectivity issues of polyether chemistries has been the use of carbonyl-containing polymers (Fig. 3c). Namely, polyesters and polycarbonates show higher ion selectivity (*t*_Li^+^_ > 0.6) to PEO owing to weaker Li-ion carbonyl *versus* oxygen ether coordination.^110,111^ However, benefits to both ionic conductivities and transference number are found in SPEs that couple both polyether and polycarbonate chemistries together.^112,113^ Subsequent refining of the poly(ether-carbonate) design would benefit from the use of controlled polymerisation strategies towards more precise placement of carbonate moieties along polyether chains. Promisingly, controlled polymer synthesis has already helped gain a better understanding of how coordination site connectivity in polyesters influences conductivities.^114^ This opens up more possibilities for the future uncovering of missing structure–property correlations.

Aside from ion transport, widening the electrochemical stability window (ESW) of polymer electrolytes is a challenge, with >4.2 V *vs.* Li/Li^+^ being an ideal case, and ultimately relies on multiple factors.^115^ Defined as the voltage range over which electrolytes are stable to oxidative and reductive degradation, the ESW affects the choice of electrode system and, thus, the theoretical specific capacities (Fig. 3g). Understanding the various complex contributions from functional groups and salt to ESW can be aided by atomic-scale modelling.^116,117^ From these studies, the reduction window appears to relate more to salt choice whereas oxidative stability depends on polymer/salt combinations. For a given salt, carbonyl- and nitrile-containing chemistries have demonstrated superior oxidative stability (Fig. 3d, 4.5–5 V *vs.* Li^+^/Li).^118,119^ The high oxidation potentials of these SPE chemistries can be correlated with the highest occupied molecular orbital (HOMO) energies.^116^ Whereas the high HOMO of PEO means it is more suited to LiFePO_4_ or LiCoO_2_ cathodes with oxidative potentials below 4 V, polycarbonates would be expected to be stable against high voltage cathodes such as LiNi_*x*_Co_*y*_Mn_*z*_O_2_ (NMC). This translates to theoretical specific capacities being limited to 170 mA h g^−1^ (*vs.* graphite) compared to 200 mA h g^−1^ with NMCs (Fig. 3g).^5^

Beyond backbone functionality, the polymer chain-end plus the use of additives can also be important in determining high-voltage compatibility along with thermal stability (*T*_d_ in Table 1).^91,107^ Some notable developments include the use of garnet-type ISE fillers to suppress oxidation,^120^ low HOMO energy plastic crystal succinonitrile additives^118^ and acetate or methoxy end-capping of –OH terminated SPEs.^92^ The rationale behind many of these strategies can be traced to the increasing recognition of the role of intermolecular interactions in SPE stability.^104^ The conversion of –OH terminated chains to trifluoroacetyl-units has a protective effect and can delay the onset of thermal decomposition by as much as 20 °C.^113^ Equally, the inclusion of incombustible groups or fillers like those based on phosphorus can be used to enhance the flame retardancy of SPEs and is another parameter to consider when designing polymers for Li anode solid-state batteries.^119^

Alongside performance merits, the sustainability of any additives, choice of electrode materials and polymer electrolyte chemistries must also be taken into account. Polycarbonate electrolytes, in particular, have attracted praise as a means of utilising CO_2_ as a waste renewable gas. This has included routes to cyclic carbonate monomer synthesis *via* CO_2_ in the case of poly(oxo-carbonate) electrolytes.^121^ Direct alternating CO_2_/epoxide copolymerisation is also a viable approach (Fig. 3f). Whereas this was discussed above for constructing mechanical modifying PC blocks, the use of alternate glycidyl ether epoxides can provide electrolytes with more honey-like consistencies and after various improvements in the catalysis, ambient conductivities of 0.01 mS cm^−1^.^122,123^ Other examples include SLICs based on appended lithium carboxylates.^124^ Importantly, an environmental impact assessment carried out by Lizundia concluded that the global warming potential is less for these CO_2_-derivable polycarbonates – they consider PPC-based electrolytes compared to others like poly(acrylonitrile) (PAN) and poly(vinylidene fluoride) (PVDF).^125^ However, the LiTFSI salt has a large impact, meaning strategies for salt recovery are essential. Additionally, the depolymerisation of many polycarbonates and polyesters has now been demonstrated, and the recovered monomer has been used to re-synthesise the polymer. This may be regarded as the most attractive option towards a closed-loop circular economy as possible degradation of the polymer electrolyte during battery operation and impurities resulting from inefficient delamination from the electrode interfaces may considerably influence the performance of the recycled polymer.^126^

Finally, the number of reports of SPEs containing fluorinated backbones, including PVDF, is worth separate consideration. Noteworthy are findings that these fluorinated chemistries can form stable passivating layers at electrode surfaces, have increased electrochemical stability and enhance salt dissociation, leading to more mobile ion concentrations and higher conductivities.^127^ However, in light of the environmental concern surrounding fluorinated materials and regulatory uncertainty, finding alternatives or strategies for recycling them must be a focus. Recent work by Xie *et al.* investigated a library of fluorinated polyester materials as solid electrolyte candidates with LiTFSI.^128^ They achieved RT ionic conductivities of 0.059 mS cm^−1^ and were able to recycle 90% of the LiTFSI and regenerate 86% of the polyester.

## 3. Opportunities for solid polymer electrolytes

The breadth of synthetic polymer chemistry offers a myriad of opportunities for the advancement of SPEs. Through meticulous control over polymer synthesis, researchers can tailor the molecular architecture, composition and functionalisation of polymers to potentially achieve the desired combination of high ionic conductivity, mechanical strength and stability properties. Polymer synthesis techniques such as controlled ROP enable the precise design of copolymers including block-type, which can facilitate efficient ion transport *via* microphase-separated structures. Additionally, novel polymer architectures, including brush, star, and dendritic polymers, offer the potential for enhanced ion transport and improved mechanical properties. In this subsection, the integration of dynamic covalent chemistry into SPEs is explored for its promise of endowing materials with self-healing capabilities and adaptability to stress and damage. Furthermore, the advantages of synthesising SPEs directly within the battery assembly *via in situ* techniques are discussed. Finally, machine learning tools are considered for their potential to propel ongoing research efforts in the discovery, design and optimisation of high-performance SPEs.

### 3.1. Dynamic chemistry – a route to ‘smarter’ polymer electrolytes

**Fig. 4:**
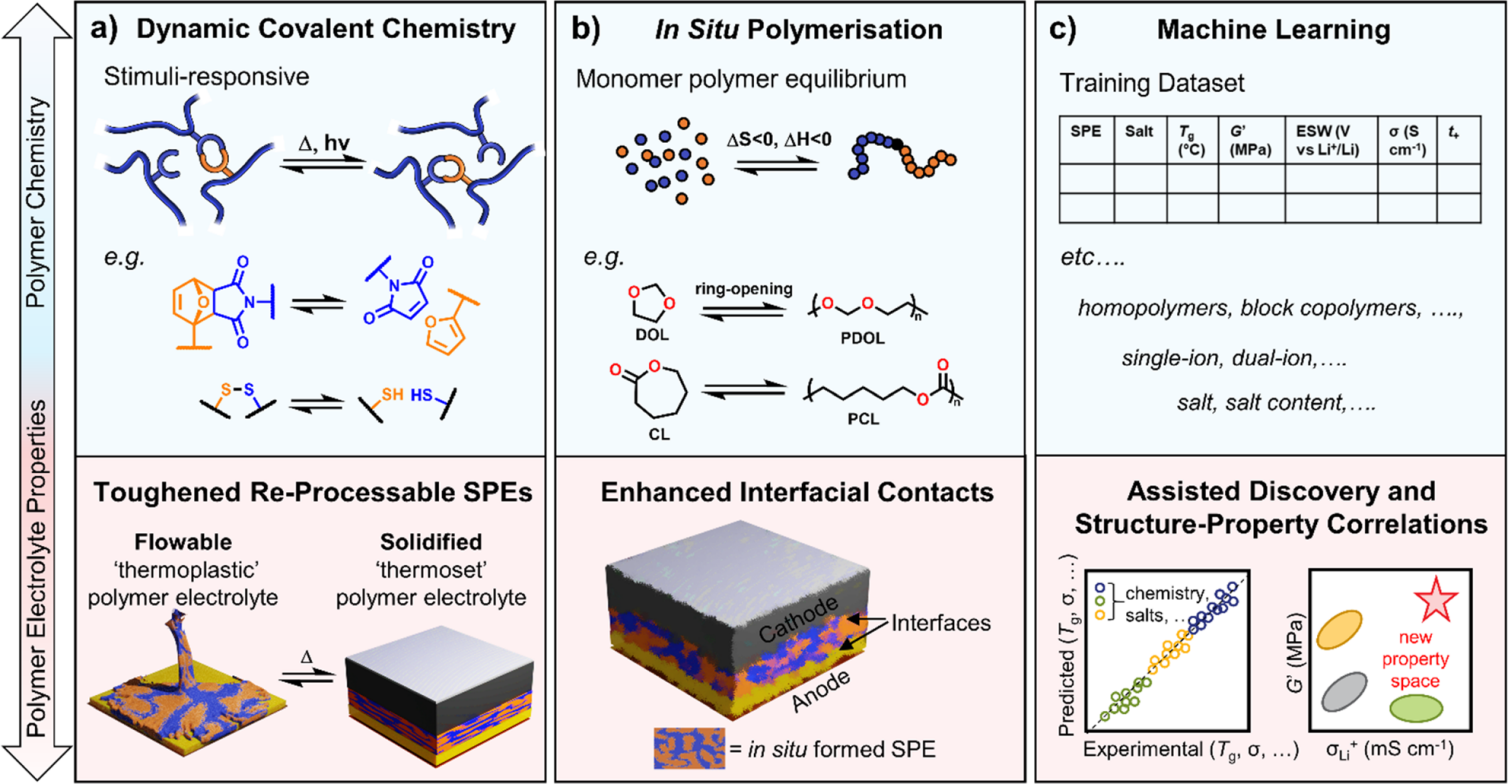
Advanced polymer electrolyte design approaches. (a) Examples of covalent bonds that can be broken and reformed under different heat (Δ) or light (*hν*) stimuli. Toughened SPEs from covalently linked networks can be rendered processable again *via* these dynamic chemistries. (b) Enthalpically-driven ring-opening polymerisation of cyclic monomers conducted *in situ* in the battery cell. (c) Machine learning models applied to SPE datasets to assist in materials discovery and the identification of missing structure–property correlations.

Dynamic polymer systems are another tool at the disposal of the polymer chemist to introduce circularity to polymer electrolyte usage.^129^ These systems invoke ‘reversible’ covalent bonds, *i.e.* those that can be broken and reformed upon the application of defined thermal and/or irradiative conditions, enabling control over polymer processing and the creation of stimuli-responsive materials (Fig. 4a).^130^ Thermosetting materials, whilst highly sought after for their robust mechanical and chemically/thermally resistant properties, lack the processability conferred by thermoplastics and can be considered ‘unrecyclable’ by design.^130–132^ The use of dynamic chemistry, specifically with covalent adaptable networks (*e.g.* vitrimers), provides a route to processable polymers with the durability of thermosets and can give rise to the recyclability of materials.^133,134^ In polymer design for batteries, robust and durable properties are needed, for example, to achieve good cycling stability. Dynamic polymers may thus find application here, including in flexible devices. This is demonstrated in select literature examples below for various dynamic functional handles.

Ion conductive and adhesive SPEs have been demonstrated using cleavable disulfide linkages. The dynamic nature of the networks stimulated through heat or UV light to break/reform the S–S crosslinks resulted in higher adhesive strength (×6) and ionic conductivities (×7) compared to the equivalent non-dynamic networks.^135^ Although the ionic conductivities were modest (10^−4^ S cm^−1^ at 90 °C), the adhesives could be debonded/rebonded with UV irradiation multiple times without loss of performance. Rearrangements of dynamic covalent disulfide bonds (alongside hydrogen bonds between urethane groups) were also recently used to construct superior electrolyte–electrode interfacial contacts in solid-state Li–S batteries.^136^ Cast directly onto the S-based and Li-metal electrodes, the self-healing poly(ether-urethane) electrolytes exhibited reduced interfacial resistance and capacity retentions of 84% over 350 cycles were reported compared to 71% with no disulfide chemistry.

Self-healing polymer electrolyte properties can also be imparted using the transamination of imine bond moieties. Using this functional handle to form/reform networked structures in cells with Li-metal and LiFePO_4_ achieved stable high 5C charging rates with discharge capacities of 118 mA h g^−1^.^137^ Importantly, after healing, SPE ionic conductivities (4.79 mS cm^−1^ at 30 °C) and mechanical strength (ability to hold a 100 kg weight) were close to the original material. Reports that the lithium salt present can also catalyse bond exchange are also of note, given this accelerates the dynamics that underpin processability and recyclability in these systems.^138^ Finally, Diels–Alder chemistry has also been used to generate thermally reprocessable single-ion conducting copolymers (*t*_Li^+^_ ∼ 1). Despite the relatively low ionic conductivities (0.07 mS cm^−1^ at 80 °C), the Diels–Alder adduct formed between the furan and maleimide moieties could be thermally reversed at 140 °C up to 30× to give polymers of comparable conductivity and mechanical robustness.^139^ Thus, the inclusion of dynamic covalent linkages is a promising strategy towards more (re)processable and toughened SPEs – potentially robust enough to suppress Li dendrite growth. They may also pave the way to depolymerisable and/or recyclable electrolytes. Given that ‘sufficient’ ionic conductivities can be achieved, this could give SPEs a competitive market edge over other materials.

### 3.2. *In situ* polymerisation for enhanced interfaces

Conventionally, polymers are introduced into cells either as powders, in slurries or as stand-alone thin films. However, forming the polymer directly in the cell (pre-cycling) by *in situ* polymerisation techniques has reported several benefits, including easier processability and enhanced interfacial compatibility compared to *ex situ* formed polymers (Fig. 4b). Although residual monomer cannot be removed *via* this strategy and may have both safety and performance implications, it can also be advantageous for ionic conductivity through self-plasticising of the polymer chains with the monomer, resulting in lower *T*_g_ and increased segmental dynamics.^140^

For example, the *in situ* polymerisation of vinylidene carbonate was possible in cells with Li metal anode and LiCoO_2_ cathode. An initial discharge capacity of 146 mA h g^−1^ was reported at 50 °C, 84% of which was retained after 150 charge/discharge cycles.^141^ This synthesis was *via* an uncontrolled free radical polymerisation and may benefit from a controlled technique to fine-tune the polymer properties. UV irradiation has also been used to induce the radical *in situ* copolymerisation of the same monomer with hydroxyethyl methacrylate to provide mechanical strength.^142^ With the inclusion of this polyacrylate segment, both an improvement in modulus (0.5 to 2.4 GPa) and ionic conductivity (0.43 to 0.8 mS cm^−1^) were observed.^142^*In situ* polymers of various acrylate monomers have also been reported using ATRP in Li-metal batteries with commercial LiFePO_4_ cathode.^143^ In these cases, the lithium salt served not only as a source of ions but also to induce the polymerisation process through activation of the ATRP initiator. This polymerisation technique, however, suffers from issues regarding scaling-up.

The ROP of cyclic lactones, carbonates and epoxides is another choice for *in situ* polymerisation owing to their high efficiency and mild polymerisation conditions.^144^ Often unfavourable entropically (Δ*S* < 0), ROP is driven by enthalpic contributions (Δ*H* < 0) related to the ring stain of the monomer (Fig. 4b). Examples include PCL electrolytes from CL and PDOL from dioxolane. Moreover, the inherent thermal equilibrium between the monomeric ring structures and the opened polymers enables chemical depolymerisation at temperatures above the ‘ceiling’ temperature where the enthalpic gain of forming a polymer chain is insufficient to outweigh the loss of entropy – catalysts have been designed to more efficiently exploit this process for polycarbonates.^145^

Towards higher capacity batteries, the sulfur-based cathodes in Li–S batteries have theoretical specific capacities of >1600 mA h g^−1^. These chemistries produce lithium polysulfide species, which can initiate *in situ* polymerisation. As the sulfide species are nucleophilic, they are incompatible with cationic polymerisation techniques but can be used in the anionic variant as an initiator. In particular, the anionic ROP of cyclic 3-membered episulfide monomers was carried out in this fashion without the need for an external catalyst. Specific discharge capacities of 505 mA h g^−1^ were recorded for some electrolyte formulations.^146^ With alternative battery types comes a different fundamental chemistry platform and new challenges to which polymer synthesis can adapt.

### 3.3. Accelerating discovery using digital learning tools

If researchers continue to target faster and more selective ion transport in polymers while also balancing mechanical and electrochemical properties, machine learning and high-throughput screening may help. Machine learning has been widely discussed as a tool to accelerate learning and predict properties (Fig. 4c).^147^ Help from machine learning may then take the form of improving current SPEs or possibly identifying previously overlooked or unknown ones. It might also allow missed or incorrectly assigned property correlations to be more quickly and efficiently identified from the underlying datasets.

Machine-learning-guided discovery of materials with high ionic conductivity and sufficient electrochemical stability window has been described.^148^ This work demonstrated machine learning as an efficient method to quickly screen for promising ionic liquids that were then fabricated with polymers as electrolyte materials in Li-metal batteries. To overcome data scarcity issues, the input features for the ionic liquids screened were from commercially available cations and anions. Clearly, there is immense potential for machine learning to be utilised in polymer electrolyte design and optimisation, but strategies to generate sufficiently large datasets are still required.

Recent evidence that reasonably sized datasets (5225 entries) could be compiled from literature data on dry SPEs (65 publications) was provided by Segalman and Seshadri.^149^ Subsequent progress, however, took SPE ionic conductivity data from hundreds of publications and trained a chemistry-informed machine learning model.^150^ Incorporating physical equations or parameters into machine learning models can enhance property prediction accuracy in the absence of sufficient data. By embedding the Arrhenius equation in their model, significantly more accurate predictions of SPE ionic conductivity based on molecular structure and composition were achieved. Over 20 000 previously unevaluated SPEs were screened based on synthetically known polymers with commonly used lithium salts. Overall, the work showcased a more streamlined approach to materials development by using machine learning to identify the most promising polymer chemistries/salt systems on which to focus experimental efforts. Given the likely larger body of published experimental data sets on SPEs, more efforts should be made to automate the extraction of these data sets using digital tools.

Machine learning may benefit the polymer electrolyte field in other, more fundamental ways. Molecular dynamics simulations of ion transport behaviour in polymers would be extremely informative to our understanding and, thus, design approach.^151^ However, these simulations are very costly due to the diversity of timescales involved in segmental chain dynamics and their preferential amorphous nature for ion conductivity. A significant reduction in this computational cost has recently been reported using assisted machine learning techniques.^151^ Whilst such advancements are exciting for the future of SPEs, there is still a way to go. For example, in developing machine learning models that can accurately predict polymer mechanical properties and how monomer sequences and more complex architectures are represented.^147^

## 4. Functional polymers in composite electrodes

While polymers have long been explored as solid electrolytes in batteries, their potential as active components within composite electrodes has more recently garnered interest in enhancing device performance. Equally, in contrast to the role of polymers as passive binders in battery electrodes, the integration of multifunctional polymers in solid composite electrodes marks a significant shift in the design of advanced solid-state batteries and wearable technologies. This subsection explores the roles that innovative, multifunctional polymers could play in addressing longstanding challenges associated with conventional binders in electrode fabrication. For instance, ionic elastic adhesive and mixed-ionic electronic conducting binders are notable advancements for potentially addressing challenges in optimising solid composite cathode microstructures. It highlights their impact on performance and durability by buffering active material volume changes and sustaining interfacial contact. By embracing the diverse approaches to polymer design, including supramolecular strategies and the early indications of promising polymeric artificial interlayers, researchers could unlock new possibilities for battery systems and tailor them to the evolving demands of modern electronics.

### 4.1. Limits of conventional polymer binders

**Fig. 5:**
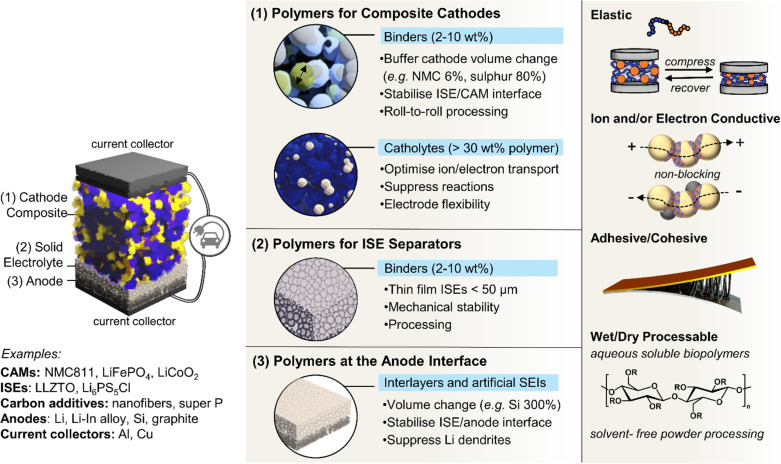
Roles of polymers in solid-state batteries with inorganic solid electrolytes (ISEs). From the left: solid-state cell with thick composite cathode comprised of cathode active material (CAM), ISE, carbon additive and polymer binder in ∼70 : 23 : 2 : 5 wt% ratio (1), thin-film ISE layer (2) and Li or Li-rich metal foil anode (3). Polymers play roles as functional binders and coatings, protective interlayers and interphases and active electrode material. SEI, solid electrolyte interphase. Elastic polymers are required to buffer electrode volume changes and intrinsic ionic and/or electron polymer conductivities to facilitate ion/electron pathways. Adhesive/cohesive polymer properties are necessary to prevent delamination failure mechanisms that shorten battery lifetime and polymer processability, which inform cell fabrication methods and, in turn, performances.

Battery electrodes are typically composite materials made up of an active material, conductive additives, and a polymer binder. The active material provides the electrochemical potential to the battery; usually, it is a transition metal oxide in the cathode and graphite, silicon, or Li metal in the anode. During discharge, Li-ions flow from anode to cathode through an electrolyte while electrons flow in the same direction *via* an external circuit – the reverse process occurs during charging. Whereas the previous sections considered the use of polymers as these electrolytes sandwiched between two electrodes, this section focuses on the importance of polymers in the electrodes themselves and at the electrolyte–electrode interfaces (Fig. 5).

In commercial liquid electrolyte-based Li-ion batteries, polymers are widely used to maintain the structural integrity of electrodes,^152^ adhere to current collectors,^153^ and form as passivating interlayers at electrode–electrolyte interfaces.^154^ Polymer function, and thus design, arguably becomes more critical as the field progresses from these liquid-based systems with graphite anodes to potentially all-solid-state and more energy-dense electrodes (Si and Li anodes, Ni-rich manganese cobalt oxides and S-based cathodes).^43,155,156^ Here, electrode volume changes are intrinsic to the lithiation/delithiation charge/discharge mechanisms and thus unavoidable. They can erode the integrity of protective interlayers and challenge the maintenance of critical interfacial contacts between active components for electron and ion flow.^31,157^

PVDF is the most commonly used binder in commercial batteries, chosen because of its high thermal, chemical and electrochemical stability. These properties also confer PVDF its environmental persistence and prompted the recent global political push away from fluorinated polymers in general.^158^ Despite its wide usage, PVDF has other drawbacks. As a poor electronic and ionic conductor, its main function is to maintain electrode integrity. Electronically conductive, usually carbonaceous, additives are added to composite electrodes to enable electrons to move from active material to current collectors and around a circuit. The addition of these materials results in lower energy density, as well as increased internal resistance as they agglomerate.^159^ Similarly, in all-solid-state devices, ISEs are typically mixed into electrode composites to provide ion pathways. This is done to mimic the natural inflow achieved by liquid electrolytes but greatly increases the tortuosity of ion pathways.^31^ PVDF or other non-ion conductive binders can further hamper these ion pathways, reducing the attainable capacity even when present in typical 2–5 wt%.^160^ Hence, the design and optimisation of a binder for any given electrode active material may be key to delivering superior batteries to meet modern demands.

The adhesive properties of PVDF and other ‘off-the-shelf’ binders are also lacking, resulting in a tendency to delaminate from cathode-active material surfaces over time – this issue is linked with the ionic conductivity problems, as both are associated with the polymer's lack of affinity for highly polar and charged species.^161^ This delamination affects the long-term cycling stability of the battery and can also be a result of inappropriate binder mechanical properties and chemical stability. When not well matched, the mechanical stresses that arise from volume changes during cycling and reactive interfaces can also lead to delamination and capacity decay.^27^

The processability of any binder must also be considered. PVDF is difficult to process, requiring *N*-methyl-2-pyrrolidone (NMP) solvent, which is expensive, significantly toxic, and reactive towards lithium metal, preventing its use as an anode.^161^ Significant progress has been made with aqueous processable binders, and in particular, most bio-polymers are water-soluble. Although this eliminates the need for toxic solvents such as NMP, using water as a solvent is incompatible with higher-capacity cathodes (such as NMCs), Li anodes, and many ISEs.^7^ Li_6_PS_5_Cl is a particularly challenging ISE owing to its high moisture sensitivity and limited compatibility with most polar organic solvents.^162^ This has led to a trend of avoiding solvents altogether in electrode fabrication and instead dry-mixing.^163^ Although potentially greener, ensuring the polymer is uniformly distributed throughout and the composite microstructure optimised for function requires additional characterisation.^7^

Given that the goal is to maximise the energy density of battery cells, this means including as much active material in the electrode as possible and thus minimising the amount of inactive material such as the binders and conductive additives. Cell designs expected to maximise capacity and energy density propose using a thin electrolyte separator (<50 μm), thick cathode composite, and Li foil anode.^164^ Delivering the necessary ultrathin ISE separators^39^ and maintaining function in thick composite cathode layers will require polymer binders.^165^ One obvious approach to lowering the mass of binder and other non-active material components required is to design more effective polymeric binders with multi-functionality. This includes elastic polymers with specific adhesive capabilities, so less is required for function, and those that are also ionically and/or electronic conductive to serve as a binder while minimising internal resistance. Of note, many of the SPEs mentioned in the previous sections and routes to confer dynamic, self-healing and enhanced interfacial contacts may also be relevant here as advanced Li-ion conducting binders.

### 4.2. Ionically conductive adhesive elastomers

Elastomeric polymers, capable of recovery after repeated compressive stress or stain, are required to buffer active material volume changes in electrode composites. Whereas commercial LiFePO_4_ cathode particles undergo negligible volume change during charging/discharging, next-generation cathode materials do. LiCoO_2_ expands by ∼2 vol% on charging and Ni-rich LiNi_0.8_Co_0.1_Mn_0.1_O_2_ (NMC811) shrinks by ∼6% upon delithiation. The latter is more desirable as it uses less Co and exhibits ∼40% higher theoretical capacity.^2,5^ The effect is more substantial in sulfur-based cathodes, which could deliver capacities ∼700% greater than the 200 mA h g^−1^ for NMC811 but undergo large volume changes of 70–80% during the reversible conversion process of S_8_ to Li_2_S_2_/Li_2_S.^166^ At the anode side, exceptionally high theoretical capacities of silicon are challenged by the extreme volume changes of up to 300% during lithiation/delithiation, making degradation especially facile and the binder even more critical.^167^

**Fig. 6:**
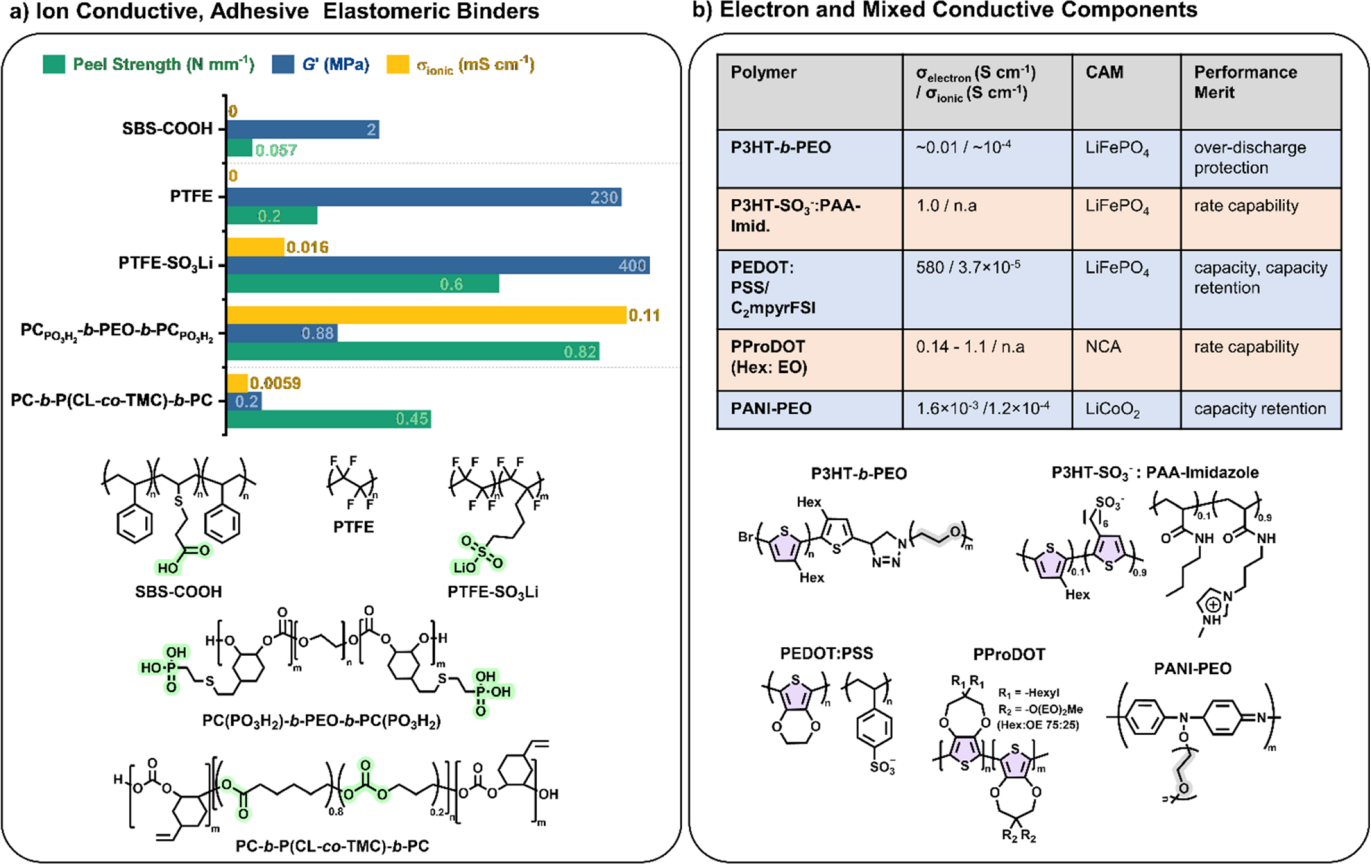
Examples of multifunctional polymer components in battery electrodes. (a) Ionic, adhesion and elastomeric binders employed in solid-state composite cathodes combining NMC cathode active material, inorganic Li_6_PS_5_Cl solid electrolyte and C. Adhesive handles are highlighted in green. (b) Ion (*σ*_ionic_) and/or electron (*σ*_e_) conductivities of conjugated polymer binders with their merit in battery performances. Blue, cell cycling with solid electrolyte (SPE or ISE); red, cell cycling with commercial liquid electrolytes; all cells use Li metal anodes. Thiophene repeat units are shaded in purple and EO in grey – these are combined with LiTFSI for ion conductivity.

In the same way that block copolymers can microphase separate to provide ion channels (Section 2.1), elastomeric polymer behaviour can be achieved by ordering minority rigid blocks in spherical or hexagonal packed cylinder arrangements within a flexible polymer matrix.^168^ Stretchability is conferred by the rubbery chains and an ability to recover by the rigid domains. A classic example is poly(styrene)-*b*-poly(butadiene)-*b*-poly(styrene), SBS, which Choi and coworkers employed to buffer volume changes in composite cathodes with Li_6_PS_5_Cl, NMC711 and C.^162^ To increase the adhesive properties and delay cell failure from delamination, they modified the polymer with hydrogen-bonding carboxylic acid groups (SBS–COOH, Fig. 6a). The extent of acid groups introduced was dictated by the need to retain solubility in non-polar Li_6_PS_5_Cl – compatible solvents for slurry-processing of the cathode. The same authors demonstrated with spandex that the shear forces during slurry mixing of elastic polymers can form coatings around NMC811 particles.^169^ These coatings were proposed to form a protective layer around the cathode particles against undesired interfacial reactions. NMC is known to react with Li_6_PS_5_Cl electrolytes, so a protective barrier was needed to prevent this.

Building upon this, Gregory *et al.* synthesised a series of elastomeric ABA-block copolymers with rigid polycarbonate (PC) ‘A’ blocks and flexible PEO ‘B’ blocks.^69^ With the addition of lithium salt and grafting of a phosphonic acid functional group on the polycarbonate blocks, these elastomers were then also ionically conductive and adhesive (PC_PO_3_H_2__-*b*-PEO-*b*-PC_PO_3_H_2__, Fig. 6a). These properties meant they had to be dry processed into composite cathodes with NMC811, L_6_PS_5_Cl and carbonaceous additives, but resulted in 23% improved capacity retention over the same cell configuration with PVDF binder. Later, the ‘B’ PEO mid-segment was replaced with a more oxidatively stable poly(caprolactone-*co*-trimethylene carbonate) (P(CL-*co*-TMC)) copolymer which enabled the use of uncoated and single crystalline NMC811 and thus access to higher capacities (Fig. 6a).^72^ As proof of principle over the lack of end-of-life options for fluorinated binders, the polymer was subsequently extracted from the cell and depolymerised to recover 90% of the starting monomers. Here, the comparison was made with PTFE rather than PVDF, the former being common for dry-processing composite cathodes as it is thought to form extended nano-fibril networks that are beneficial to electrode integrity.^170^ There is no evidence that the polycarbonate-ester-based alternative formed similar structures during dry mixing. Nevertheless, the Li-ion conducting polymer/LiFSI system outperformed PTFE in terms of capacity (186 *vs.* 177 mA h g^−1^) and capacity retention (97 *vs.* 86% after 200 cycles).

A more direct comparison of switching from non-conductive PTFE binder to an even modestly ion-conducting version (0.016 mS cm^−1^ at 25 °C) was reported using the lithium sulfonate single-ion-conducting derivative (PTFE–SO_3_Li, Fig. 6a). In a similar cell configuration to above 5 wt% of the cathode composite binders showed vastly improved cycling performance for the single-Li-ion-conducting version over its non-conductive analogue (90 *vs.* 24% capacity retention over 300 cycles).^171^ In fact, more than the ionic conductivity, the high performance of the single-ion conductor could be attributed to enhanced contact between the cathode active material and electrolyte particles afforded by the charged sulfonate groups. Hence, this study highlighted the importance of not only ionic conductivity but also interfacial adhesion afforded by binders in cell performance.

Together with the further design of adhesive, elastomeric, and ionically conductive multifunctional binders, experiments with active electrode materials that undergo larger volume changes are needed. In these cases, polymer design may be expected to have an even greater impact on performance. For example, sulfur-based cathodes have benefited from mechanically responsive networks comprised of polyaniline (PANI) and PEO.^172^ Aside from mitigating volume changes, inhibiting the detrimental shuttle of polysulfides formed in Li–S batteries is a key target; the latter showed good trapping ability of these species. Single-ion conductors based on lithium borates have also shown promise as both ion transporters and polysulfide-trapping binders in Li–S battery chemistries.^88^

On the anode side, Nature's polymers, polysaccharide derivatives, are naturally oxygen-rich and have high densities of hydrogen bonding capability. This gives them unprecedented adhesive qualities that have proven attractive for adhering to the complementary hydrophilic silicon dioxide surfaces on Si anodes. To this end, various groups have explored the use of carboxymethyl cellulose (CMC) sodium and lithium salts, as well as sodium alginate from algae, as moderately ionically conducting (∼10^−4^ S cm^−1^) yet biodegradable binders.^173^ Bio-based binders are usually water-processable, which leads to poor compatibility with most cathode materials other than LiFePO_4_, but they are appropriate for use in graphite and Si anodes. To sufficiently buffer the large Si volume changes, blending of these binders with poly(styrene-*b*-butadiene) rubbers is required. In this respect, gum arabic – a polysaccharide and glycoprotein mixture is promising as the glycoproteins act as mechanically reinforcing fibres, obviating the need for additional rubber additives.^174^

Although natural polymers have disadvantages in terms of the security and reproducibility of supply, these studies highlight fundamental polymer properties that inform synthetic binder design. Of note, CMC binders have been adopted for commercial use in graphite anodes, demonstrating a successful shift away from PVDF to a superior, more sustainable polymer at market level. Future research targets might seek to improve upon the molecular design offered by nature through synthetic sugar-based mimics.^175^ These also offer more scope for tailoring functionality to improve ion transport properties and flexibility. Self-healing Li-ion conductive polyurethanes and biodegradable urea cross-linked PCL have also been used to promising effect with Si anodes.^176^ Again, these benefit from hydrogen bonding between the urethane or urea groups and elastomeric properties from combining these rigid moieties with soft polymer segments.

### 4.3. Mixed ion-electron conducting polymeric components

Electronic conductivity is essential in electrode materials to facilitate electron transfer between the active materials and the current collectors. Typical composite electrodes use carbonaceous substances such as acetylene black or carbon nanofibers (2–10 wt%) to improve electronic conductivity. These can agglomerate over many charge–discharge cycles, leading to an increase in internal resistance and concomitant drop in capacity.^159^ By introducing electronic conductivity (*σ*_e^−^_) into the electrode binders, the internal resistance is reduced, and a lower quantity of carbonaceous additives is required, allowing for higher active material loadings. Highly electronically conductive binders have been shown to significantly improve the capacity of some cathodes (*e.g.* LiNi_0.8_Co_0.15_Al_0.05_O_2_, NCA) when cycling at high rates, meaning these binders could have an impact on fast-charging battery technology.^177^

Most electronically conductive polymers are semiconductors with extended conjugated pi systems; their conductivity is low (10^−10^ to 10^−5^ S cm^−1^) in the neutral state but improves significantly on doping (up to 10^5^ S cm^−1^). As conjugated polymers tend to be hydrophobic, their ionic conductivities and adhesion with polar electrode active materials are usually poor; however, decorating them with polar or charged side chains can improve both properties.^177^ It has been proposed that polymers with electronic and ionic conductivity exhibit a synergistic effect that enhances both transport mechanisms.^178^ Accessing mixed ionic-electronic conductors in polymer binders is clearly a useful target where optimisation of both conductivity pathways in composite electrodes is essential.

There has been significant recent interest in using polythiophene derivatives to introduce electronic conductivity into polymer binders (Fig. 6b). In particular, the properties of poly(3-hexylthiophene) (P3HT) have been well studied, paving a significant basis for exploration as electrically conducting binders in composite electrodes. Their low band gaps make them effective conductors upon doping electrochemically or with chemical dopants. For example, Balsara and coworkers reported P3HT-*b*-PEO binders.^179^ During battery charging, the P3HT blocks were oxidised, enabling reasonable electronic conductivity (10^−4^ S cm^−1^) during discharging due to holes forming in the valence band. Towards the end of the discharge cycles, the P3HT was reduced back to the neutral state, dropping the electronic conductivity by three orders of magnitude to an essentially insulating state. This phenomenon aided in preventing over-discharging in the cell.^179^ Similarly, the groups of Tolbert and Dunn showed that P3HT doped with carbon nanotubes (20 wt%) can serve as a coating for NCA cathodes, displaying the same effect of preventing over-discharging.^180^ Furthermore, the coating improved capacity retention by over 400% compared to PVDF after 1000 cycles at high 16C cycling rate and formed a solid-electrolyte interlayer that protected the breakdown of the NCA active material.

Following this, blends of poly(3,4-ethylenedioxythiophene) and poly(styrene sulfonate) (PEDOT:PSS) are popular commercial mixed-ion-electron conducting polymers. The sulfonate groups (–SO_3_^−^) in the PSS component enable ion transport and blends of PEDOT:PSS have exhibited electronic conductivities of the order of 4.7 S cm^−1^. As binders in Si anodes, these values were sufficiently conductive to eliminate the need for carbonaceous additives, allowing for high Si loadings (up to 95 wt%).^181^ Similarly, they could also be used C-free in LiFePO_4_ cathode composites where they were applied with ionic plastic crystals and cycled in Li anode cells with a solid polymer electrolyte at 70 °C. Capacities of 145 mA h g^−1^ (0.5C) were achieved with excellent capacity retention (99.7% after 500 cycles).^182^ These findings suggest that mixed polymer conductors are a key technology for future use in batteries.

PEDOT itself (without PSS) has also shown promise as a cathode coating material for Li–S batteries. It was used to maintain the structure of the sulfur cathode particles, which are highly susceptible to degradation over time. PEDOT demonstrated superior cycling efficiency compared to polypyrrole (PPy), PANI, and PVDF coatings. However, capacity fade was still observed.^183^ While PEDOT is an effective electronic conductor, its ionic conductivity remains low, and poly(3,4-propylenedioxythiophene) (PProDOT) has proved useful in providing tuneable side-chain functionalities to enhance ionic conductivities. Initial work used hexyl side chains to enable processability and swelling in liquid electrolytes.^184^ Carbon nanofibres were included in this electrode, and the overall effect resulted in up to 5× improved coulombic efficiency when charging at 6C compared with PVDF binder. Subsequent work improved upon this by replacing some of the hexyl chains with very short PEO chains (2 EO units).^185^ Although cycling was improved, >50% PEO content resulted in dissolution into the battery electrolyte. Future research might prevent this using a solid-state electrolyte.

Most recently, the Segalman group reported electrostatically stabilised blends of anionic P3HT (P3HT–SO_3_^−^) with oppositely charged polyelectrolyte (imidazole polyacrylamide) as processable, mixed conducting and C-free binders for LiFePO_4_ cathodes (Fig. 6b).^186^ They reported that the multifunctional binder improved rate capability and capacity retention compared to PVDF (63% over 400C/2 cycles *vs.* 6%). Complexation of the charged P3HT by the polyelectrolyte was proposed to template the P3HT conformation, leading to improved electronic conductivity from 0.001 to 1 S cm^−1^.

While polythiophenes have traditionally been synthesised *via* ‘Grignard metathesis’, the modern development of direct heteroatom-arylation polymerisation (DHAP) has been applied in recent years to perform greener and more atom-efficient polymerisations.^187^ This is promising for the future commercialisation of polythiophene-based technologies. There are also examples of *in situ* polythiophene synthesises using electro-polymerisation strategies^188^ and a recent report of degradable conjugate polymer backbones *via* cleavable imine bonds.^187^ Such opportunities for controlled and *in situ* approaches alongside end-of-life options bring conjugated polymers more on par with the controlled polyester/carbonate synthesises above, where the polymerisation conditions are mild and ester/carbonate bonds are readily hydrolysable.

Building upon these studies with polythiophene-based mixed conductors, recent developments have demonstrated the potential of flexible dual conducting polymers in the cathode composite of Li anode solid-state batteries with solid electrolyte (Li_6.4_La_3_Zr_1.4_Ta_0.6_O_12_, LLZTO/PVDF binder).^189^ By integrating PANI polymers grafted with PEO side chains (PANI–PEO, Fig. 6b) into the LiCoO_2_ cathode composites of these batteries, a substantial increase in cycling stability was observed compared to no polymer cathodes (92 *vs.* 6% capacity retention). The flexible nature of these polymers contributes to the better accommodation of volume charges during charge–discharge cycles and the dual conduction facilitates uniform and fast ion and electron transport. The polymer is also reported to stabilize the generation of cathode interface layers. Consequently, these advancements offer a promising pathway towards more durable and efficient next-generation solid-state batteries *via* polymer design modifications of cathode composites.

### 4.4. Self-healing supramolecular polymers

Whilst supramolecular polymer chemistry is still relatively underexplored in the context of battery binders, Coskun and Choi have developed several promising examples based on supramolecular principles involving cyclodextrin macrocycles.^190^ In this work ‘hyperbranched’ copolymers of cyclodextrin with epichlorohydrin were found to adhere well with Si anodes due to their high hydrogen bonding capacity and the branched structure, enabling more entanglement with the anode particles. As a result, significantly less contact loss occurred upon the large decrease in volume on Si delithiation compared to linear (non-branched) analogues. Furthermore, these interactions enabled recovery of contact, leading to a desirable self-healing effect in the composite anode. The neat β-cyclodextrin polymer showed nearly double the capacity retention of linear polymers. However, this was still only about half after 150 cycles. Subsequent work by the same group introduced a ‘guest crosslinker’ to the system.^191^ The crosslinker contained six branches capable of forming strong guest–host interactions with the hyperbranched β-cyclodextrins-based polymers. The resulting mixture formed a dynamic as well as self-healing cross-linking binder with a remarkably more efficient capacity retention of up to 90% after 150 cycles.

Another approach threaded many α-cyclodextrin units onto PEO chains, which were then grafted to poly(acrylic acid) to form a cross-linked polyrotaxane polymer system. The grafted cyclodextrin rings acted as ‘pulleys’ for the PEO ‘rope’, which aided in the dissipation of stress within the system – a film of the polymer did not rupture until 390% strain.^192^ The high elasticity of this topological system enabled an outstanding capacity retention of 98% after 50 cycles in a Si anode against NCA cathode. Overall, these findings support the benefits of thought-out polymer design in battery applications and the potentially promising future of supramolecular chemistry in this area.

### 4.5. Engineering protective artificial interlayers

Electrolytes can react at the surface of electrode materials to form beneficial solid-electrolyte interphases (SEIs). In Li anodes, SEIs can passivate the reactive Li-metal surface, preventing further degradation.^193^ Hence, the ability to form stable SEI layers can be a determining factor in cell performance fading, even in cells with solid electrolytes and especially with Si anodes.^193^ Consequently, because of this and the potential opportunity to minimise interfacial resistance and dendrite growth further, researchers have become interested in designing artificial SEI layers. Engineering these layers has so far proved challenging, however, owing to the spontaneous manner in which they form and through both chemical and electrochemical reactions, resulting in difficulties in characterising their exact nature and composition. Recent significant progress has been through advanced solid-state NMR studies, and typically, beneficial SEI layers are complex mixtures of oligomers, polymers, and lithium salts.^194^

Whereas ceramic coatings have been used as artificial SEIs in the past, cracking with volume changes led to the loss of function with cycling. The flexibility of polymers lends itself to accommodating the repeated volume changes without cracking or loss of contact. Artificial SEI manufacturing is still in its infancy. However, the application of design principles from polymer electrolytes and binders has springboarded the field in artificial SEI designs for batteries with both current liquid and future solid electrolytes. Recent work demonstrated excellent cycling performance with single-ion conductive cross-linked interlayers at the Li anode/polymer electrolyte interface (86% capacity retention after 1000 cycles at 1C; 76% at 2C).^195^ Blends of fluorinated and ionically conducting polymers have also demonstrated the formation of passivating layers at the Li metal – garnet-type oxide ISE interface. These showed remarkable long-term capacity retention of 98% after 110 cycles at 0.5C, in contrast to rapid degradation of the anode in the absence of the SEI layer. The fluorine-rich polymers reacted with the metal anode to form a LiF layer, which aided in uniform lithium deposition.^196^ Evidently, these reports suggest that SEIs could have important value in the future of Li metal batteries for both safety and capacity retention. Future research avenues might focus on the use of ionically conductive elastomers for applications with different electrolyte systems and approaches to eliminating fluorinated polymers from the SEI layer.

## 5. Polymers for wearable technologies

The integration of polymers in wearable technologies is a convergence of material science and electronics, catering to the growing demand for flexible, lightweight and functional devices. Building upon the advancements in SPEs and functional polymer binders in solid-state battery composite electrodes outlined in the preceding sections, the focus now shifts to polymer design to meet the requirements of wearable devices where flexibility, biocompatibility, and sustainability are paramount. This section presents the polymers shaping the landscape of wearable technologies, particularly in the development of flexible polymeric electrodes, biodegradable polymer substrates, and encapsulation materials. It offers insights into their applications and implications for the future of electronics worn on the body.

### 5.1. Battery requirements: energy density, flexibility and safety

**Fig. 7:**
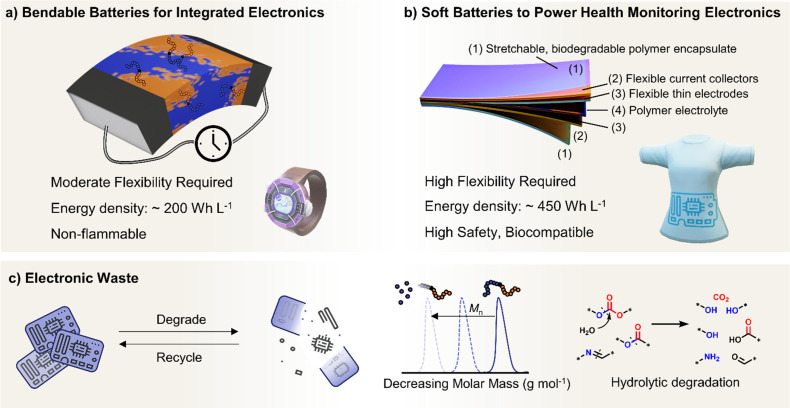
Polymer considerations for wearable technologies. (a) Bendable, conformable batteries to power smartwatches; (b) soft, skin-interfaced devices. (c) End-of-life of battery components; depolymerisation and/or (bio)degradation of polymeric materials.

Polymer design for batteries must also face the challenges of emerging wearable batteries, which are at the core of wearable electronics. Zhi categorised wearable batteries into two types: (1) those integrated into the electronics like smartwatches and foldable phones (Fig. 7a); (2) those intimately attached to the body for healthcare monitoring such as ‘smart’ eye masks or clothing (Fig. 7b).^48^ The energy density, flexibility, and safety requirements for each type are different. To power wearables like smartwatches, the battery space available is increasingly limited as devices become more and more compact and lightweight. Hence, energy density is a critical component of these batteries to satisfy battery life. Enhancements may require even newer battery chemistries and accompanying polymer designs to those discussed above.^47^

These wearable technologies and thus their in-built battery electronics are also increasingly desired to be bendable to conform to our body shapes. This requires development and innovation in all the battery components to prevent delamination from one another and, thus, performance issues as the whole system deforms.^197^ The safety of these devices, though extremely important, is not as stringent as that of wearables that interface with the human skin directly. These types, for health monitoring, are typically integrated polymer semi-conductors. They require lower energy densities, but battery thicknesses are reduced as much as possible to achieve high flexibility and conformability, as well as adhere to strict safety considerations for skin bio-compatibility.^46,48^

With flexibility an integral component of wearable technologies, polymer mechanical properties are a good fit. Non-flammable, solvent-free polymer-based electrolytes are highly attractive for addressing the combined aspects of safety and flexibility. However, until the aforementioned issues with the ionic conductivity of SPEs are resolved, quasi-solid or gel polymer electrolytes may be the best choice today.^198^ Where liquids are employed, aqueous-based electrolytes are preferable to flammable organics, though using liquids significantly limits the attainable energy densities.^45^ The electrodes themselves also need to be suitably flexible as does the battery casing or encapsulant layer. For maximum device softness and multi-dimensional deformability, all-polymer-based battery chemistries are potentially beneficial. However, significant challenges remain.

### 5.2. Flexible polymeric electrodes

The fabrication of flexible electrodes is a necessary step towards flexible batteries for wearables. Given that active electrode materials are traditionally stiff (
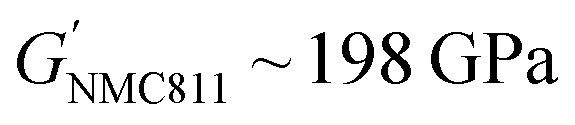
; 
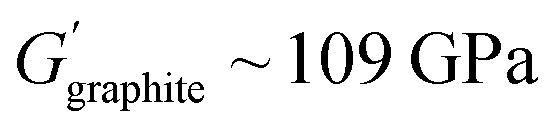
), flexible alternatives like bendable polymer matrices are required.^46^ The interfacial adhesion between the electrode and current collectors (flexible C papers/fabrics as opposed to rigid Al/Cu foil) also needs to be strong and highly durable to resist delamination during bending.^197^ In the previous section, we considered the properties of polymer binders in composite electrodes (2–10 wt%) to buffer volume changes.^197^ Here, we discuss the polymer as the bulk electrode component (>40 wt%) to meet the extra demands for mechanical stability under high deformation.

Polymer active materials can replace inorganics to fabricate flexible and thin composite electrodes for wearables. These can form either the anode or cathode, but most commonly, they are employed as cathode active materials in combination with Li or Zn metal anodes.^48^ All-polymer-based batteries have polymer active materials as both electrodes, and depending upon the redox potential of the specific polymers, typical cell voltages are 1–2 V. Polyacetylene was first used as a rechargeable cathode material by MacDiarmid and Heeger in 1981;^199^ since then, a wide variety of conjugated polymers have been applied as electrode materials, particularly polypyrroles, polythiophenes, and polyanilines.^200^ Typically, conjugated polymers have high voltage potentials but significantly lower capacities than state-of-the-art anode and cathode active materials, although the associated mechanical and processing advantages may outweigh the loss in energy density for low-power devices. One benefit of conjugated polymer-based electrodes is that they typically do not require carbonaceous additives due to their high conductivities (up to 1000 S cm^−1^), which also enables super-fast charging. However, flexible batteries employing these electrodes can be prone to self-discharging and poor cycle life.^200^

Radical polymers with TEMPO side chains and various backbones have also been investigated. Most notable in terms of performance are those from the cationic polymerisation of vinyl ethers (PTVE—poly(2,2,6,6-tetramethylpiperidinyloxy-4-yl-vinylether))^201^ and the anionic ROP of glycidyl ethers (PTEO—poly(4-glycidyloxy-2,2,6,6-tetramethylpiperidine-1-oxyl)).^202^ Both these polymerisation strategies are compatible with the presence of radical centres. The theoretical specific capacity for PTVE is 110 mA h g^−1^, but for PTEO, it is much higher at 220 mA h g^−1^.^203^ The TEMPO radicals can undergo both oxidation and reduction with high potentials (>3 V) and are used as cathode materials, although, in principle, could also find use in anodes.^46^ These materials offer high current density and can be charged at high C-rates (up to 100C). They do, however, require carbon additives due to low intrinsic electrical conductivities and can have issues with self-discharge. A promising prospect of these types of polymer chemistries is their potential as 3D printable electrode materials^204^ and reported syntheses from biomass.^205^ Attachment of the radical pendants to polypeptide backbones has also been shown by Wooley *et al.* to enable on-demand degradation for recyclability and towards a sustainable battery economy.^206^

Recent work in polymeric anode materials has explored ‘super-lithiation’, where carbon-rich species are lithiated at many or all unsaturated positions, enabling improved capacity. Unsaturated polymers, including polypyrroles, polyimides, and pyrene-fused azaacene polymers, have all been super-lithiated for use as anode materials with far higher specific capacities than graphite (up to 1900 mA h g^−1^).^207^ The loss of unsaturation during super-lithiation results in a loss in conductivity, meaning conductive additives are again required for super-lithiated polymer anodes.

Polymers as electrode materials offer unique opportunities for molecular design to impart mechanical properties that cannot be achieved with traditional inorganic electrode materials. Strategies such as block copolymerisation and conjugation-breaking spacing can modify flexibility and stretchability. The introduction of additional side chain functionalities can improve the ionic conductivity and processability of polymer electrodes. Focus on new polymer electrodes should be directed towards preventing self-discharge and improving their capacities for use in flexible high-power devices.

### 5.3. Biodegradable polymer substrates and encapsulants

For mechanical deformation, wearable technologies usually require electrochemically inactive substrates and encapsulation layers. Previously, we have ignored any consideration of the housing or outermost shell of the battery. In wearable batteries, particularly for health monitoring, the encapsulation layer is a defining feature to device flexibility and human interfacing.^208^ Using a solid electrolyte lessens the requirement for these layers also to contain and prevent dangerous liquid electrolyte leakage or evaporation, leading to flammability concerns. This subsequently allows the use of thinner, softer and more deformable encapsulant layers.^48^

Several research efforts have been made to use biodegradable elastic polymers as encapsulation layers to introduce the desired flexibility and compatibility with human skin.^209^ Like the polymer requirements for elastic binders (Section 4.2), these encapsulation layers require even higher performing mechanical properties, including flexibility, elasticity, and resistance to fracture over repeated motions. Many studies have employed block copolymer elastomers as stretchable substrates for electrodes and encapsulants.^210^ Similarly, oxygen-rich polymers (polyesters, carbonates) are beneficial for skin adhesion, and many are synthesised from naturally oxygen-rich bio-derived monomers (lactones, cyclic carbonates).^211^ These polymers have greater bio-compatibility potential plus prospects for biodegradability at end-of-use. Fitting both properties together, there are several examples of block copolymer elastomers made from bio-derived monomers. These include examples of all-polyester triblock copolymers synthesised from bio-derived monomers like ε-decalactone from castor oil.^168^ The use of controlled polymerisation techniques (*Ð* ∼ 1.1, *M*_n_ > 100 kg mol^−1^) allowed easy manipulation of the block ratios to achieve desirable high 98% elastic recovery, tuneable elastic moduli ranging from 1 to 10 MPa and high stretchability (>1000% strain). Although the modulus of skin is softer (on the order of kPa), there are various other examples of degradable polyester elastomers that could be used as insulating encapsulants, including those employing dynamic covalent crosslinking (Section 3.1)^212^ and based on copolymers of biodegradable/bio-based polycaprolactone and polylactide.^209^

Apart from targeting biocompatibility, the use of bioderived and degradable polymer materials feeds into concepts of transient electronics.^49^ As electronic devices have become ubiquitous in today's society and increasingly single-use, there is an accompanying accumulation of electronic waste (Fig. 7c). To address this, polymer chemists need to design and advocate for their components in batteries and wearable technologies to be efficiently reusable or safely absorbable into the environment at end-use. Fortunately, many of the polyester/carbonate chemistries that are attractive for ion transport (Section 2.3) and *in situ* polymerisation (Section 3.2), plus the dynamic covalent chemistries (Section 3.1), are also amenable to depolymerisation strategies, breakdown *via* hydrolysis and reprocessing.^211^ Conjugated polymer backbones (Sections 4.3 and 5.2) are intrinsically not susceptible to hydrolysis. The synthesis of biodegradable, electronically conductive materials remains a challenge in polymer chemistry, though there is evidence of significant and ongoing progress in this area.^187^

## 6. Conclusions and outlook

### 6.1. Structure–property relationships to unlock ion transport

State-of-the-art solid polymer electrolytes still typically deliver room temperature ionic conductivities *ca.* an order of magnitude below liquid electrolytes and the best inorganic solid electrolytes. Several factors are important for polymer ion conductivity, such as the impact of glass transition, segmental dynamics, viscosity and fragility. Many of these, in turn, are controllable by polymer *M*_n_, dispersity, chemistries and architecture. Opportunities exist, however, to further understand and identify/address knowledge gaps, for example, in single-ion conductors and synergies between conductivity mechanisms. Dynamic covalent chemistry and *in situ* polymerisation techniques could make polymer electrolytes more competitive, at least as recyclability and processing requirements become dominant. Polymers are also especially poised for the requirements for wearables, and it is hoped that digital learning tools could help crack ion transport issues faster. Broadly, whether room temperature polymer performances are still the most critical target should also be discussed. This is given local heating in battery environments, and the impact functional polymers are beginning to show in enabling inorganic solid electrolyte-based batteries.

### 6.2. Consistency for performance evaluation

Polymer chemical, electrochemical, and thermal stability are paramount in the battery environment. Polymer mechanical performances, processability, adhesive/cohesive strength, and transport properties (conductivity and selectivity) are also essential. Evaluating these properties in a reliable, consistent and meaningful way is thus extremely important. For example, electrochemical stability windows should be measured by linear sweep and cyclic voltammetry against sensible electrode materials and at slow scan rates (<0.1 mV s^−1^) in line with the slow kinetics of solid-state degradation reactions. A couple of methods exist to measure ion selectively or transference numbers, and each can produce a different result for some systems. Adhesion and cohesion can be difficult to evaluate comparably across the field by standard peel test measurements. It can also be hard to ensure adhesion to all component materials in a composite electrode, especially when some inorganics are highly air and moisture-sensitive.

### 6.3. Translation and integration into operating devices

After designing a library of potentially suitable polymer materials and measuring their properties, it is critical to understand how these then translate into battery performances. How measured polymer parameters connect with device performance is important not only for iterative polymer synthesis to improve performances but also for a better understanding of the problem. This requires a highly interdisciplinary and collaborative approach between polymer and battery scientists. Equally, more detailed reporting of ‘of-the-shelf’ polymer components (*M*_n_, *Ð*, *etc.*) used in published device data is needed. Designing polymeric components to optimise performances in next-generation batteries will require tailoring to each of the various battery chemistries and other specific active materials present. Future research may also see an increase in reporting on processing effects – this can have a significant impact on aspects like binder distribution in composite electrode microstructures, which influence battery operation. Processability is also increasingly a hurdle to the commercialisation of new battery chemistries and an important parameter in evaluating their sustainability credentials.

### 6.4. Preparing for beyond lithium and ultra-bendable technologies

Unanswered questions remain on lithium dendrite suppression with single-ion, self-healing polymeric electrolytes, the limiting factors of polymer–inorganic electrolyte interfaces (important for composite electrodes and hybrid electrolytes) and how to achieve binder distributions for optimal composite electrode microstructures. Polymer chemists should prepare for beyond lithium-based batteries and devices. There are a rapidly growing number of reports on polymeric sodium-ion conductors, but K(i), Ca(ii), Mg(ii) *etc.* are also potentially promising. Early findings support that the ion transport of these larger radii and multivalent ions will differ from Li(i). Future research might focus more on the metal–ligand coordination chemistry to fine-tune properties with these higher valent ions.

### 6.5. Balancing sustainability and performance

Perhaps counterintuitively, polymers required to be stable in highly demanding, harsh battery environments should also offer end-of-life degradability and be sourced from renewable, ideally bio-derived monomers. The environmental concerns of fluorinated polymers go somewhat hand-in-hand with their chemical inertness and common choice for battery applications. Conversely, polycarbonates are susceptible to acid and base hydrolysis but are widely revered for their high oxidative stability. Of course, most high-performance batteries rely on components that necessitate anhydrous environments, and any water present to hydrolyse carbonate moieties would already be detrimental to the cell. End-of-life strategies, such as depolymerisation, biodegradation, and recycling, should not be overlooked. The question of which option is the best and the most efficient strategy may depend on the cell configuration, other components in the cell, and their relative quantities.

## Author contributions

F. L. and K. S. contributed to the curation and analysis of the literature as well as the planning and writing of the manuscript. G. G. supervised and directed the work, wrote the manuscript with F. L. and K. S. and designed the figures.

## Conflicts of interest

There are no conflicts to declare.
